# Facial Lichen Planopilaris Mimicking Melasma: A Case Report Emphasizing the Value of Clinicopathologic Correlation

**DOI:** 10.1155/crdm/1703880

**Published:** 2026-06-28

**Authors:** Maryam Hekmat, Negin Fazelzadeh Haghighi, Fatemeh Sari Aslani, Farideh Jokar

**Affiliations:** ^1^ Molecular Dermatology Research Center, Shiraz University of Medical Sciences, Shiraz, Iran, sums.ac.ir; ^2^ Department of Dermatology, Shiraz University of Medical Sciences, Shiraz, Iran, sums.ac.ir; ^3^ Department of Pathology, Shiraz University of Medical Sciences, Shiraz, Iran, sums.ac.ir

**Keywords:** facial hyperpigmentation, lichen planopilaris, lichen planus, melasma

## Abstract

Lichen planopilaris (LPP) is a rare, immune‐mediated cicatricial alopecia characterized by perifollicular erythema, hyperkeratosis, and progressive hair follicle destruction, leading to permanent hair loss. While LPP typically affects the scalp and is classified into classic LPP, frontal fibrosing alopecia (FFA), and Graham‐Little–Piccardi–Lassueur (GLPL) syndrome, atypical presentations involving extra‐scalp regions, including the face, are exceedingly rare. We present a unique case of melasma‐like LPP manifesting on the face, a highly unusual presentation that posed diagnostic challenges due to its resemblance to hyperpigmentary disorders. A 38‐year‐old man presented with an asymptomatic hyperpigmented patch on the cheek and forehead mimicking melasma. This case highlights the importance of considering LPP in the differential diagnosis of facial hyperpigmentation, particularly when conventional therapies for melasma fail. It should be noted that histopathology was a valuable tool for the diagnosis of the disease. We recommend including dermoscopic images in future studies to help dermatologists become familiar with the dermoscopic features of LPP and to differentiate between LPP and melasma using dermoscopy.

## 1. Introduction

Lichen planopilaris (LPP) is a variant of lichen planus which causes immune‐mediated cicatricial alopecia [1]. It is a rare disease of the scalp which causes alopecia in 1.25% of patients [2]. It presents as itchy, multifocal patches with perifollicular erythema and follicular hyperkeratosis. In dermoscopy, perifollicular erythematous and violaceous papules, as well as spinous follicular hyperkeratosis, support the diagnosis of LPP [3]. Lichen planopilaris of the face is an uncommon form of the disease, with only a limited number of cases documented in the scientific literature [4, 5]. The underlying cause of this condition remains unknown, and it is distinguished by the presence of pigmented follicular papules that gradually develop into atrophic lesions [5]. Melasma is a condition of acquired hyperpigmentation marked by light to dark brownish macules or patches that typically appear on sun‐exposed skin, most commonly on the face [6]. Therefore, melasma and LPP have different presentations. In this report, we describe a case of LPP with melasma‐like presentation on the cheek and forehead, which is not typical for LPP.

## 2. Case Description

**FIGURE 1:**
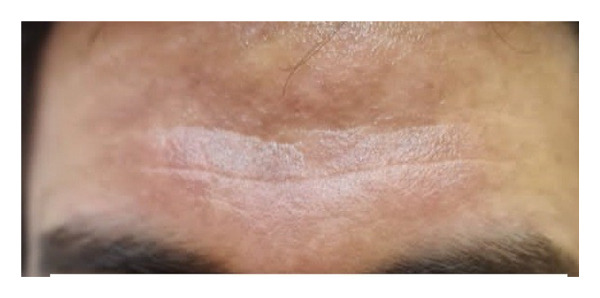
Melasma‐like red‐brown patches in the central part of the face.

A 38‐year‐old man presented to Shahid Faghihi Dermatology clinic at Shiraz University of Medical Sciences with a history of an asymptomatic hyperpigmented patch on the cheek and forehead. Physical examination showed a melasma‐like, red‐brown patch in the central part of the face (Figure 1). The rest of the skin, oral mucosa and nails were normal. The patient was otherwise healthy.

**FIGURE 2:**
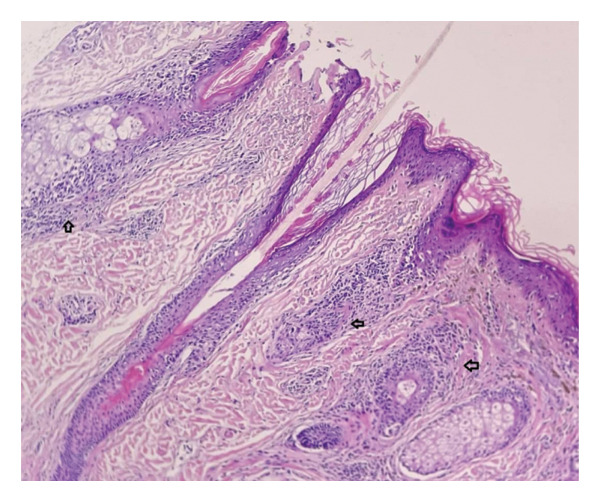
Mild hyperkeratosis, dilated infundibulae, basal cell degeneration of mainly infundibular epithelium with hypergranulosis, perifollicular lymphocytic infiltration (black arrows), and pigment incontinence in the papillary dermis (H&Ex100).

**FIGURE 3:**
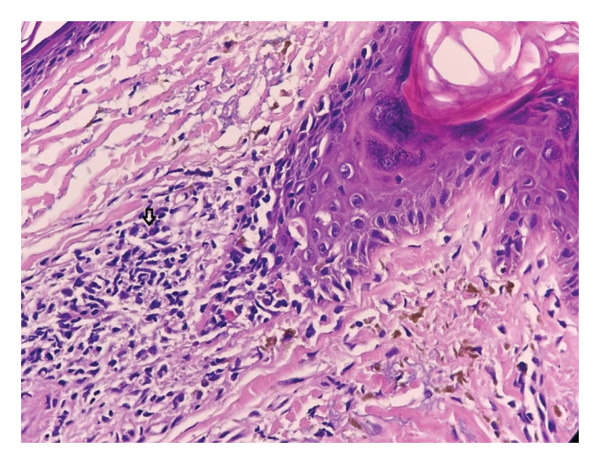
Dilated infundibulum, basal degeneration of mainly infundibular epithelium with civatte body formation, hypergranulosis, peri‐infundibular lymphocytic infiltration (black arrow), and pigment incontinence in the papillary dermis (H&Ex400).

Dermoscopy showed follicular erythema and pigmentation. Microscopic examination showed mild hyperkeratosis, dilated infundibulae with few Demodex mites, basal degeneration of mainly the infundibular epithelium with hypergranulosis, perifollicular mild to moderate lymphocytic infiltration, sparse perivascular inflammation, and pigment incontinence in the papillary dermis and peri‐infundibular area. No deep dermal or perieccrine inflammation was seen (Figures 2 and 3). The diagnosis of melasma‐like LPP of the face was confirmed.

The patient received topical tacrolimus 0.1% and oral hydroxychloroquine 200 mg twice daily. After 6 months, he had partial improvement in his skin lesion.

## 3. Discussion

The case presented in this article describes a patient with a melasma‐like facial presentation, which was diagnosed through histopathology as having a lichenoid reaction with feature of LPP.

LPP is a follicular form of lichen planus which affects women more than men. The typical age of onset is 40–60 years, and the exact etiology of the disease is unknown [7]. Although traditionally LPP is subdivided into three variants—classic LPP, frontal fibrosing alopecia (FFA), and Graham‐Little–Piccard–Lassueur syndrome (GLPL)—other rare variants have been reported. These variants include scarring alopecia of the vulva, lichen planus follicularis tumidus, and linear LPP of the face. An atypical case of facial LPP was reported in 2013, describing a 46‐year‐old man who presented with asymptomatic facial umbilicated papules [4]. Chloe Goldman et al. also reported a case of scalp LPP presenting as reticulated hyperpigmentation [8]. The primary presentation of some patients with FFA is lichen planus pigmentosus (LPPi). In 2014, the first case report of LPPi with FFA was described, and then, a case series of 24 African patients was published [9]. LPPi is an important differential diagnosis for facial and melasma‐like lesions and should be considered in facial hyperpigmentation. Al‐Husain KM et al. reported a melasma‐like LPPi case caused by Vicks VapoRub (VVR), which is an inhalant ointment used for the alleviation of upper respiratory tract infections [10].

Histopathology is considered a valuable tool to differentiate these melasma‐like diagnoses, as the common histologic features of LPPi are pigment incontinence, vacuolar degeneration of the basal cell layer, dermal and perivascular lymphocytic inflammation, and thinning of the epidermis [11]. In the literature, some reports have also described patients with melasma‐like actinic lichen planus [12, 13]. Therefore, one of the probable differential diagnoses of our case was actinic lichen planus. In histopathology, actinic lichen planus mostly shows interface dermatitis with vacuolar degeneration of the basal cell layer, mid‐dermal perivascular lymphocytic inflammation, and pigment incontinence [14]. The case we presented, however, was histopathologically different from these two entities, as the infiltration, which is predominantly observed in the late stages, is a diagnostic characteristic of LPP on histopathology [15]. Ultimately, another differential diagnosis of our case was melasma which is an acquired hypermelanosis of the skin characterized by irregular brown macules and patches on the face. Histologically, it is characterized by epidermal hyperpigmentation with hypertrophy of melanocytes, while LPP shows lymphocytic inflammation around the hair follicle [15, 16]. It should be noted that dermoscopy is another tool that can help dermatologists to differentiate these melasma‐like lesions mentioned above. On dermoscopic examination for melasma, prominent hyperpigmentation in the pseudoridges of the skin can be detected. It presents as dark‐brown, light brown, and bluish‐gray patches when melanin is located in the stratum corneum, lower epidermal layers, and dermis respectively [6]. Dermoscopic features of LPP include keratotic follicular papules, perifollicular scales and erythema, and arboriform vessels. Whitish or milky‐red areas and absence of follicular opening can be seen at the advanced stages of the disease. Dermoscopic examination of LPPi usually reveals dark brown to gray dots and globules in various patterns, including dotted, speckled, diffuse, reticular, and circular. Erythema and telangiectatic vessels are sometimes noted [17]. All melasma‐like actinic LP patients show annular granular patterns, dots inside circles and pigmentation around circles on dermoscopic examination. Other features that can be seen include reticular pigmentation and perifollicular hypopigmentation [18]. According to the abovementioned differential diagnoses for melasma, we should consider other diseases in cases with melasma, particularly in refractory cases. Refractory melasma is defined as unresponsiveness to treatments for more than 6 months [19]. Melasma‐like presentation of LPP has not been reported in the literature until now. Therefore, this case is worthy of reporting due to its unique presentation.

### 3.1. Limitations

The limitation of the current study was that we did not provide the dermoscopic images of the patient in this article. We recommend including dermoscopic images in future studies to help dermatologists become familiar with the dermoscopic features of LPP and to differentiate between LPP and melasma using dermoscopy.

## Author Contributions

Maryam Hekmat involved in clinical diagnosis of the case, literature review, and writing the manuscript. Negin Fazelzadeh Haghighi involved in literature review and writing the manuscript. Fatemeh Sari Aslani involved in pathological confirmation of the diagnosis and writing the manuscript. Farideh Jokar involved in clinical diagnosis of the case, treatment, and writing the manuscript.

## Funding

The authors have nothing to report.

## Ethics Statement

The study was approved by the Ethics Committee of Shiraz University of Medical Sciences (IR.SUMS.REC.1403.177).

## Consent

The patient signed written informed consent to permit us to use his photograph and publish the article. The personal information of the patient was kept confidential.

## Conflicts of Interest

The authors declare no conflicts of interest.

## Data Availability

All data are available in the main text.
